# Differential effects of long‐term slow‐pressor and subpressor angiotensin II on contractile and relaxant reactivity of resistance versus conductance arteries

**DOI:** 10.14814/phy2.13623

**Published:** 2018-03-04

**Authors:** Phillip G. Kopf, Laura E. Phelps, Chad D. Schupbach, Alan K. Johnson, Jacob D. Peuler

**Affiliations:** ^1^ Department of Pharmacology Midwestern University Downers Grove Illinois; ^2^ Departments of Psychological and Brain Sciences Health and Human Physiology, and Pharmacology the University of Iowa Iowa City Iowa

**Keywords:** Nitric oxide, nitroprusside, norepinephrine, slow‐pressor dose of Ang II

## Abstract

Vascular reactivity was evaluated in three separate arteries isolated from rats after angiotensin II (Ang II) was infused chronically in two separate experiments, one using a 14‐day high, slow‐pressor dose known to produce hypertension and the other using a 7‐day low, subpressor but hypertensive‐sensitizing dose. There were three new findings. First, there was no evidence of altered vascular reactivity in resistance arteries that might otherwise explain the hypertension due to the high Ang II or the hypertensive‐sensitizing effect of the low Ang II dose. Second, the high Ang II dose exerted a novel differential effect on arterial contractile responsiveness to the sympathetic neurotransmitter, norepinephrine, depending on the level of sympathetic innervation. It clearly enhanced that responsiveness in the sparsely innervated aorta but not in small mesenteric resistance arteries or the proximal (conductance) portion of the caudal artery, both of which are densely innervated. This suggests that the increased expression of alpha adrenergic receptors after long‐term exposure to Ang II as previously reported for aortic smooth muscle, is prevented in densely innervated arteries, likely due to long‐term Ang II‐mediated increase in sympathetic neural traffic to those vessels. Third, the same high dose of Ang II impaired aortic relaxation in response to the nitric oxide (NO) donor nitroprusside without impairing aortic endothelium‐dependent relaxation. NO is the main relaxing substance released by aortic endothelium. Accordingly, it is possible that this dose of Ang II is also associated with enhanced release of and/or enhanced smooth muscle responsiveness to other endothelial relaxing substances in a compensatory capacity.

## Introduction

It is commonly known that angiotensin II (Ang II) acts on multiple targets in the pathogenesis of human essential hypertension (Johnson et al. 2015), including direct effects on the vasculature as well as indirect effects mediated through the central nervous system (CNS). Direct effects induced by long‐term systemic infusion of exogenous Ang II at hypertensive doses include marked vascular hypertrophy of large conduit arteries (aorta, superior mesenteric, femoral) and related increases in their contractile responses to various vasoconstrictor agents in general (Jennings et al. 2010). We and others have demonstrated that hypertension induced long‐term by lower, but still slow‐pressor doses of exogenous Ang II consists of two distinct phases (Fink et al. 1987; Cox and Bishop 1991; Gorbea‐Oppliger et al. 1994; Xue et al. 2012a). Over the first few days, the rise in arterial pressure depends largely on a direct arterial constrictor action of the Ang II itself. But this is soon replaced by a second phase in which the hypertension is sustained and appears to be neurogenic in nature. There is evidence that this phase is due at least in part to a centrally mediated increase in peripheral sympathetic nerve activity (Severs and Daniels‐Severs 1973; Luft et al. 1989; Cox and Bishop 1991; Xue et al. 2012a). However, that does not rule out the possibility of increased cardiovascular responsiveness to the sympathetic neurotransmitter, norepinephrine, particularly at the level of small systemic resistance arteries which play a major role in the regulation of arterial pressure. If the chronic infusion of the slow‐pressor dose of Ang II (e.g., 120 ng/(kg·min) for 14 days) is preceded by exposure to a low subpressor dose (10 ng/(kg·min) for 7 days followed by a 7‐day delay), which by itself does not affect pressure, then the rise in arterial pressure during the long‐term neurogenic phase of the high, slow‐pressor Ang II is accelerated (Xue et al. 2012a). Whether this hypertensive‐sensitizing effect of such low, nonpressor Ang II is also associated with altered vascular contractile reactivity to norepinephrine (or Ang II itself) has not been determined. In addition, there is considerable current interest in the ability of long‐term systemic administration of Ang II to differentially alter factors that determine availability of and responsiveness to nitric oxide (NO) and other vascular relaxant substances in the arterial wall, independent of its ability to alter blood pressure (Mollnau et al. 2002; Hilgers and Webb 2007; Dal‐Ros et al. 2009; Giachini et al. 2011; Crassous et al. 2012; Broekmans et al. 2016). Accordingly, the goal of this study was to evaluate vascular reactivity to both contractile and relaxant substances after a high hypertension‐eliciting dose of Ang II and after a low nonpressor dose of Ang II, and in three separate arteries selected specifically for their unique resistance versus conductance vascular properties.

## Materials and Methods

### Animals

Male Sprague–Dawley rats (10–12 weeks old) were obtained from Envigo (Indianapolis, IN) and housed in temperature‐ and light‐controlled quarters with free access to rat chow and water ad libitum as described previously (Xue et al. 2012a). All experiments were conducted in accordance with the National Institutes of Health Guide for the Care and Use of Laboratory Animals and were approved by the Animal Care and Use Committee of Midwestern University.

### Experimental treatments

There were two experiments conducted in this study, both in which rats were implanted subcutaneously under brief isoflurane anesthesia with osmotic minipumps (Alzet) for infusion of either Ang II or its vehicle (normal saline). In the first of these two experiments, rats were infused for 14 days with a high, slow‐pressor (hypertensive) dose of Ang II (high Ang II: 120 ng/(kg·min)) in parallel with other rats infused with the vehicle, both at a delivery rate of 0.5 *μ*L/h (minipump model 2002). These rats were then euthanized for removal of experimental tissues as described below. In the second experiment, rats were infused for 7 days with a low subpressor, yet hypertensive‐sensitizing, dose of Ang II (low Ang II: 10 ng/(kg·min) in parallel with other rats infused with the vehicle, both at a delivery rate of 1 *μ*L/h (minipump model 2001). After this 7‐day delivery, euthanasia as described below for removal of experimental tissues was delayed for another 7 days to ensure that any of the infused low‐dose of Ang II was completely metabolized for reasons described previously (Xue et al. 2012a).

### Experimental tissues

At the end of each of the above experimental treatment periods, each rat (one Ang II‐treated and one vehicle‐treated per day) was weighed and then euthanized under carbon dioxide to allow removal (into cold physiological buffer) of the heart and the following intact arterial segments: the middle portion of the thoracic aorta, the most proximal portion of the ventral caudal artery, and a collection of several third‐order arterial resistance branches of the superior mesenteric circulation that perfuses the small intestine. After removing atria from each heart, the remaining ventricles were weighed (wet) and the ventricular‐to‐body weight ratio was calculated. After removing surrounding fat and connective tissue from each of the arterial segments, they were cut into intact cylindrical rings of uniform size. For rings from the aortic and caudal arterial segments, this was achieved by cutting them with a bound set of evenly spaced scalpel blades which produced rings of identical length (3‐mm each). Each of these aortic and caudal rings was then mounted between two tungsten wire stirrups, which in our experience, are thin enough (100 microns in diameter each) not to damage their inner endothelial cell layers (Peuler et al. 2012). Each third‐order mesenteric branch was cut into a ring equal to the length of the gap (2‐mm) of the myograph support system used to mount it between two even thinner (25 *μ*m) tungsten wires (620M Multi Wire Myograph System, Danish Myo Technology, Aarhus, Denmark).

Each vascular ring was then suspended in an organ bath containing physiological buffer (in millimolar units: 130 NaCl, 15 NaHCO_3_, 4.7 KCl, 1.2 KH_2_PO_4_, 1.6 CaCl_2_, 1.2 MgSO_4_, 5.5 glucose, 0.026 EDTA). The buffer was warmed to 37°C and gassed to pH 7.4 with regulated delivery of 95% oxygen/5% carbon dioxide. They were then allowed to equilibrate at resting (loading) tensions for at least 30 min before beginning evaluation of their vascular reactivity. For aortic and caudal rings, tensions were recorded using Grass instrument force transducers attached to a Grass paper chart recorder. Their resting tensions were set at 18 and 15 mN, respectively, which in preliminary tests maximized contractile tensions induced by exposure to a high potassium (K^+^) buffer. For mesenteric resistance arterial rings, tensions were recorded using a wire myograph system (Danish Myo Technology, Aarhus, Denmark). Their resting tensions were normalized to achieve 90% of the internal circumference at which those tissues would have been if relaxed and under a transmural pressure of 100 mmHg (0.9**•**IC100), as previously recommended (Mulvany and Halpern 1977).

### Vascular reactivity tests

After each arterial ring was fully equilibrated (i.e., remained stable at its designated resting tension), it was exposed twice to buffer containing a high, maximally effective contractile concentration of K^+^ (60 mmol/L). In preliminary tests, more than two exposures typically did not produce contractions higher than the second. These responses served to confirm ring contractile viability, to normalize contractions induced by Ang II and norepinephrine (to percent of maximal K^+^ response) and to calculate submaximal contractions with phenylephrine as required below. After obtaining the K^+^ contractions, each ring was returned to buffer with normal K^+^ (6 mmol/L) and then tested for vascular reactivity to cumulative administration of multiple graded concentrations of the following agents: Ang II followed by norepinephrine for contractile responses, and acetylcholine (ACh) followed by nitroprusside for relaxant responses. Between each test, each ring was washed repeatedly with agent‐free normal buffer until it returned to and remained stable at or near its designated resting tension (readjusted to that tension if necessary) before beginning administration of the next agent. To measure ACh‐induced (endothelium‐dependent) and nitroprusside‐induced (endothelium‐independent) relaxations, each arterial ring was first precontracted with phenylephrine at concentrations adjusted in an attempt to yield submaximal levels of tension near or slightly above 50% of the maximal high K^+^‐induced contractions as described above and as previously recommended (Shimokawa et al. 1996). These relaxations were then expressed as % relaxation of the phenylephrine‐induced precontractions.

### Analysis of data

Data were analyzed for statistically significant differences using unpaired t‐tests or analysis‐of‐variance (ANOVA) followed by multiple mean comparison tests as appropriate. Differences were considered statistically significant if the standard probability of error (*P* value) was less than 0.05.

## Results

Table 1 shows effects of high and low subcutaneous infusions of Ang II on cardiac ventricular weights and related ventricular‐to‐body weight ratios. Infusion of the high dose of Ang II significantly increased both measures (compared to vehicle infusion) while infusion of the low dose failed to influence either. This is consistent with several of our previous studies in which the same high Ang II produced sustained hypertension (Xue et al. 2012a,b, 2014, 2016a,b; Yu et al. 2013, 2015) accompanied by similar increases in ventricular weight measures (Yu et al. 2013), whereas the low (subpressor) dose did not (Xue et al. 2012a, 2014; Clayton et al. 2014).

**Table 1:** Body and cardiac ventricular weights for adult male rats after subcutaneous (sc) infusion of either a high slow‐pressor dose of angiotensin II (high Ang II: 120 ng/(kg·min) vs. saline vehicle at 0.5 *μ*L/h for 14 days) or a low subpressor yet hypertensive‐sensitizing dose (low Ang II: 10 ng/(kg·min) vs. saline vehicle at 1 *μ*L/h for 7 days followed by a 7‐day delay)

Weights	14‐day sc infusion		7‐day sc infusion	
	Vehicle	High Ang II	Vehicle	Low Ang II
BW (g)^a^	366 ± 6	369 ± 5	368 ± 7	369 ± 6
VW (mg)^b^	1142 ± 21	1250 ± 24^c^	1137 ± 29	1172 ± 36
VW/BW (mg/g)	3.12 ± 0.05	3.39 ± 0.07^c^	3.09 ± 0.05	3.18 ± 0.07

Values are expressed as mean ± SEM (*n* = 10 each).

a BW, body weight.

b VW, ventricular weight.

c *P* < 0.05 vs. 14‐day vehicle (unpaired *t*‐test).

**Figure 1:**
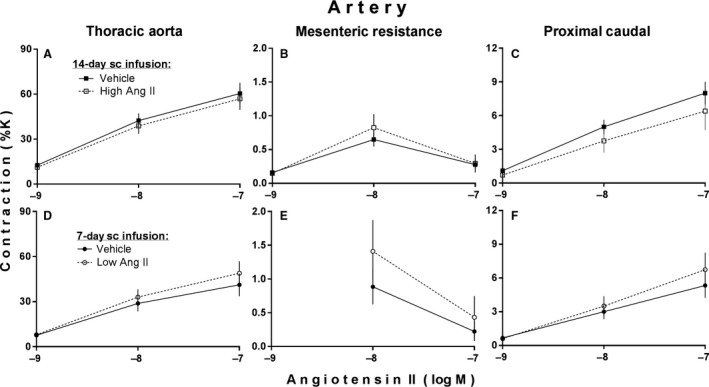
Angiotensin II‐induced contractions of arteries isolated from adult male rats after subcutaneous (sc) infusion of either a high slow‐pressor dose of angiotensin II (high Ang II:120 ng/(kg·min) versus saline vehicle at 0.5 *μ*L/h for 14 days; (A–C) or a low subpressor yet hypertensive‐sensitizing dose (low Ang II:10 ng/(kg·min) versus saline vehicle at 1 *μ*L/h for 7 days followed by a 7‐day delay; D,E,F). Values are expressed as % of maximal K contraction (%K) and illustrated as mean ± SEM (*n* = 10 each).

Figure 1 shows effects of high and low subcutaneous infusions of Ang II on vascular contractile reactivity to Ang II itself. There was a notable tendency for the high Ang II infusion to decrease and the low Ang II infusion to increase contractile responses to the direct in vitro administration of Ang II to proximal caudal arterial rings (Fig. 1C and F), but this contrasting effect did not achieve statistical significance. There were no noteworthy and/or significant differences in vascular reactivity to Ang II in any of the other two vascular preparations (i.e., thoracic aortic and mesenteric resistance arterial rings). In addition, as seen previously in mesenteric arterial tissue in general (Fasciolo and Binia 1981), our mesenteric resistance arterial ring preparations exhibited considerable tachyphylaxis in response to Ang II itself. This is evidenced in the form of markedly smaller contractile responses to the highest in vitro test concentration of Ang II compared to contractile responses produced by the concentration administered immediately prior to it (Fig. 1B and E). An attempt to minimize such tachyphylaxis by omitting administration of the lowest of the three test concentrations (in the second of the two experiments) was unsuccessful (Fig. 1E). However, although highly variable the contractile responses to the remaining two test concentrations were still large enough to allow adequate statistical evaluation of the effects of the Ang II infusion in that experiment.

**Figure 2:**
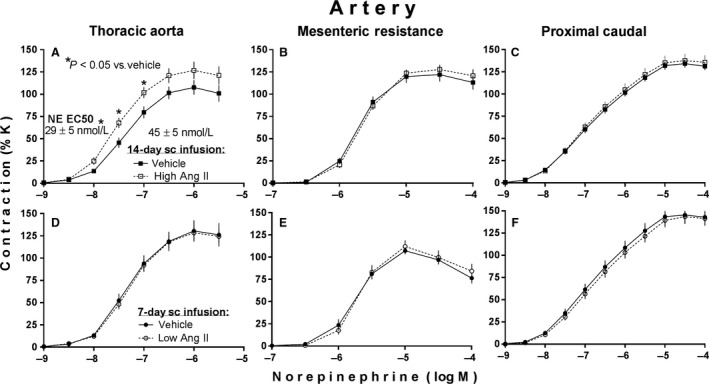
Norepinephrine (NE)‐induced contractions of arteries isolated from adult male rats after subcutaneous (sc) infusion of either a high slow‐pressor dose of angiotensin II (high Ang II:120 ng/(kg·min) versus saline vehicle at 0.5 *μ*L/h for 14 days; (A–C) or a low subpressor yet hypertensive‐sensitizing dose (low Ang II:10 ng/(kg·min) versus saline vehicle at 1 *μ*L/h for 7 days followed by a 7‐day delay; (D,E,F). Values are expressed as % of maximal K contraction (%K) and illustrated as mean ± SEM (*n* = 10 each).

Figure 2 shows effects of high and low subcutaneous infusions of Ang II on vascular contractile reactivity to norepinephrine. Infusion of the high dose of Ang II significantly increased the magnitude of contractile responses to norepinephrine in thoracic aortic rings (Fig. 2A) as determined by two‐factor ANOVA (and subsequent multiple mean comparisons) in which factor 1 = high Ang II versus vehicle infusion and factor 2 (repeated measures factor) = the multiple test concentrations of norepinephrine. Consistent with this effect, the high Ang II infusion also significantly decreased the half‐maximally effective concentration (EC50) values for norepinephrine in the same aortic rings (Fig. 2A) as determined by unpaired t‐test. Such effects were not seen in either the mesenteric resistance or proximal caudal arterial rings isolated from the same animals (Fig. 2B and C). Subcutaneous infusion of the low dose of Ang II did not influence contractile reactivity to norepinephrine in any of the arterial vascular ring preparations isolated from those animals (Fig. 2D, E and F).

**Figure 3:**
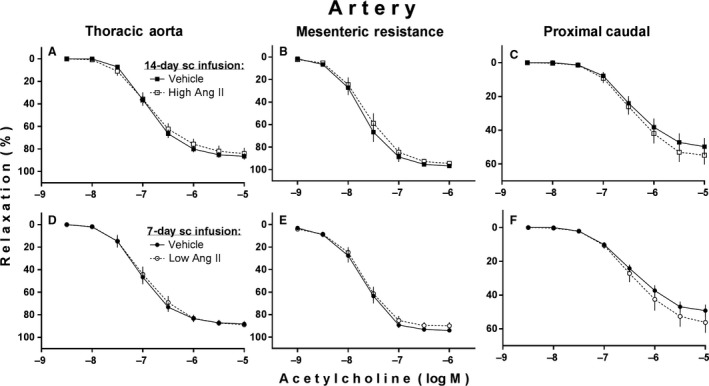
Acetylcholine‐induced (endothelium‐dependent) relaxations of phenylephrine‐precontracted arteries isolated from adult male rats after subcutaneous (sc) infusion of either a high slow‐pressor dose of angiotensin II (high Ang II:120 ng/(kg·min) vs. saline vehicle at 0.5 *μ*L/h for 14 days; (A,B,C) or a low subpressor yet hypertensive‐sensitizing dose (low Ang II:10 ng/(kg·min) versus saline vehicle at 1 *μ*L/h for 7 days followed by a 7‐day delay; (D,E,F). Values are illustrated as mean ± SEM (*n* = 10 each). Phenylephrine precontractions in mN units were (vehicle vs. Ang II): (A) 11.2 ± 0.4 vs. 12.3 ± 1.0; (B) 10.1 ± 1.1 vs. 8.7 ± 0.9; (C) 29.1 ± 2.2 vs. 28.5 ± 2.7; (D) 10.3 ± 0.9 vs. 10.8 ± 0.8; (E) 11.8 ± 0.6 vs. 10.6 ± 1.0; (F) 24.5 ± 2.3 vs. 25.8 ± 2.1.

Figure 3 shows effects of high and low subcutaneous infusions of Ang II on vascular endothelium‐dependent relaxations to ACh. There were no statistically significant effects of either infusion on ACh‐induced relaxations in any of the arterial vascular ring preparations. There were also no statistically significant effects of either infusion on the magnitude of phenylephrine‐induced precontractions required to perform the ACh‐induced relaxation tests (data in Fig. 3 legend).

**Figure 4:**
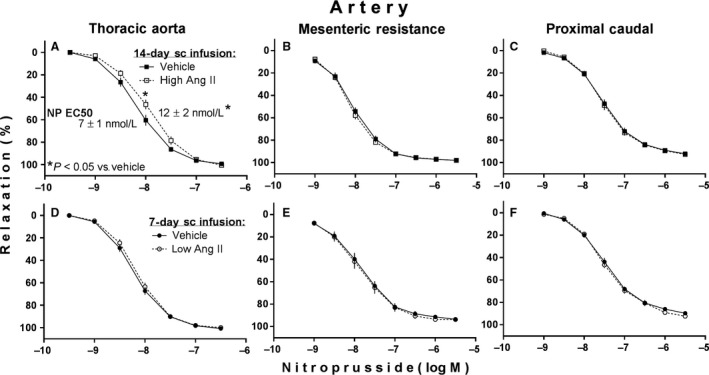
Nitroprusside(NP)‐induced (endothelium‐independent) relaxations of phenylephrine‐precontracted arteries isolated from adult male rats after subcutaneous (sc) infusion of either a high slow‐pressor dose of angiotensin II (high Ang II:120 ng/(kg·min) vs. saline vehicle at 0.5 *μ*L/h for 14 days; (A,B,C) or a low subpressor yet hypertensive‐sensitizing dose (low Ang II:10 ng/(kg·min) vs. saline vehicle at 1 *μ*L/h for 7 days followed by a 7‐day delay; (D,E,F). Values are illustrated as mean ± SEM (*n* = 10 each). Phenylephrine precontractions in mN units were (vehicle vs. Ang II): (A) 11.8 ± 0.5 vs. 13.0 ± 0.9; (B) 9.1 ± 0.8 vs. 7.8 ± 0.9; (C) 30.5 ± 1.7 vs. 31.1 ± 2.3; (D) 11.6 ± 0.7 vs. 11.9 ± 0.4; (E) 11.5 ± 0.6 vs. 10.6 ± 1.1; (F) 28.0 ± 2.4 vs. 29.5 ± 2.0.

Figure 4 shows effects of high and low subcutaneous infusions of Ang II on vascular endothelium‐independent relaxation to the NO donor nitroprusside. Infusion of the high Ang II significantly decreased the magnitude of relaxant responses to submaximal concentrations of nitroprusside in thoracic aortic rings (Fig. 4A) as determined by two‐factor ANOVA (and subsequent multiple mean comparisons) in which factor 1 = high Ang II versus vehicle infusion, and factor 2 (repeated‐measures factor) = the multiple test concentrations of nitroprusside. Consistent with this effect, the high Ang II infusion also significantly increased the half‐maximally effective concentration (EC50) values for nitroprusside in the same aortic rings (Fig. 4A) as determined by unpaired *t*‐test. Such effects were not seen in either the mesenteric resistance or proximal caudal arterial rings isolated from the same animals (Fig. 4B and C). Subcutaneous infusion of the low Ang II did not influence relaxant reactivity to nitroprusside in any of the arterial vascular ring preparations from those animals (Fig. 4D, E and F). There were also no statistically significant effects of either infusion on the magnitude of phenylephrine‐induced precontractions required to perform the nitroprusside‐induced relaxation tests (data in Fig. 4 legend).

## Discussion

### New Findings

In this study, vascular reactivity was evaluated in three separate arteries with distinctly different properties isolated from adult rats after long‐term infusion of Ang II at two different doses. There are a number of important new findings. First, intact vascular rings isolated only from the thoracic aorta following 14 days of Ang II infused at a dose known to induce hypertension displayed increased contractility in response to norepinephrine. Second, the same aortic rings showed impaired relaxation in response to the NO donor nitroprusside, but yet, unimpaired relaxation to ACh which is known to release NO from aortic endothelium. Third, vascular reactivity of third‐order mesenteric resistance vessels and the proximal portion of the caudal artery was not affected by the same 14‐day Ang II pretreatment. Finally, when infused for 7 days, a much lower dose of Ang II that does not induce hypertension by itself but is known to sensitize hypertensive responses to subsequent challenges, produced no maintained changes in vascular reactivity.

### Infusion of high Ang II and enhanced vascular reactivity to norepinephrine

The pressor response produced by systemic administration of Ang II is partially mediated by actions of the peptide on the CNS (Buggy et al. 1977). Accordingly, hypertension produced by such Ang II appears to be sustained over the long term at least in part by a centrally mediated increase in peripheral sympathetic nerve activity (Severs and Daniels‐Severs 1973; Luft et al. 1989; Cox and Bishop 1991; Xue et al. 2012a) involving action of blood‐borne Ang II with the subfornical organ (Hendel and Collister 2005) and/or the area postrema (Ferrario 1983; Fink et al. 1987; Cox and Bishop 1991). However, as suggested in the Introduction, this does not rule out a possible contribution from an increased peripheral responsiveness to the sympathetic neurotransmitter, norepinephrine, particularly at the level of the resistance vessels which regulate arterial pressure. Up to now, efforts to assess that contribution using isolated vessels have yielded conflicting results (Wang et al. 2006; Hilgers et al. 2007; Giachini et al. 2011) Our findings might resolve such conflicting results by demonstrating that there are differential effects of long‐term systemic administration of high Ang II in various vascular beds, which likely depend on their degree of sympathetic innervation.

The most important new finding from this study was the enhanced contractile responsiveness to norepinephrine in intact arterial rings prepared from rat aorta but not from small mesenteric resistance or proximal caudal arterial tissues after 14 days of subcutaneous infusion of the high dose of Ang II (120 ng/(kg·min); Fig. 2). Norepinephrine contracts smooth muscle by activating alpha adrenergic receptors, the most abundant of which in smooth muscle of aorta and most other arteries are the alpha‐1 subtypes (Civantos Calzada and Aleixandre De Artinano 2001). Several hours of exposure of cultured rat thoracic aortic smooth muscle cells to Ang II can markedly increase the number of such receptors and the transcriptional rate of their gene expression as evidenced by increased mRNAs (Hu et al. 1995). There is also evidence that over a period of days, endogenous Ang II has the same effect in vivo. For example, 7 days of captopril administration to inhibit production of endogenous Ang II, has been shown to decrease both mRNA expression and the contractile function of alpha‐1 receptors in thoracic aortic smooth muscle of young prehypertensive rats (Godinez‐Hernandez et al. 2006).

Despite this evidence that Ang II can upregulate alpha receptors in vascular smooth muscle, an increased contractile response to norepinephrine was only observed in one of the three vascular tissue preparations that we tested, that is the aorta. One potential explanation for this differential effect could be the extent of sympathetic innervation. The aorta and most other conductance arteries are only sparsely innervated (if at all in aorta of rat vs. other species) (Patil et al. 1972; Kuchii et al. 1973), whereas most resistance arteries are densely innervated (Spector et al. 1972; Burnstock 1975; Cowen and Burnstock 1980; Nilsson 1985). One notable exception amongst conductance vessels is the proximal portion of the rat ventral tail artery, which has an unusually dense sympathetic innervation (Cheung 1982; Sittiracha et al. 1987) thought to play a role in centrally directed, sympathetically mediated thermoregulation (Smith et al. 1998). Otherwise, its properties more closely resembles large conduit arteries (Souza et al. 2008).

Thus, the degree of sympathetic innervation could be a contributing factor in the pathophysiology of Ang II‐induced vascular changes in alpha adrenergic receptors. It is well‐known that chronic exposure of peripheral adrenergic receptors to increased sympathetic nerve activity and related high concentrations of norepinephrine can lead to their downregulation and/or desensitization (Snavely et al. 1983; Deighton et al. 1988; Hogikyan and Supiano 1994; Nishikawa et al. 1994; Lamba and Abraham 2000; Seals and Dinenno 2004; Westcott and Segal 2013), which at least for alpha‐1 receptors is the exact opposite of the above mentioned effect of Ang II. Therefore, we hypothesize that the increased sympathetic tone that occurs with long‐term high Ang II infusion (Severs and Daniels‐Severs 1973; Luft et al. 1989; Cox and Bishop 1991; Xue et al. 2012a) may result in a compensatory downregulation of alpha adrenergic receptors in densely innervated vascular tissues (e.g., mesenteric resistance arteries and proximal tail artery), antagonizing any direct upregulating effect of such Ang II on those same receptors. Accordingly, since the rat aorta is largely devoid of sympathetic innervation (Patil et al. 1972; Kuchii et al. 1973), only the direct effect of Ang II on its alpha adrenergic receptor expression would be present, which would then result in an increased expression of those receptors, leading to the increased contractile responsiveness to norepinephrine that we observed. Extensive future efforts will be needed to fully test this overall hypothesis. It was not feasible to do so in this study.

This hypothesis is supported by two previous studies in isolated branches of mesenteric arteries other than the third‐order branches which we employed. In these other ex vivo studies, contractile responsiveness was not increased to either norepinephrine or the more alpha‐1 selective agonist phenylephrine in second‐order branches isolated from mice or in fourth‐order branches isolated from rats after 14 days of Ang II infused subcutaneously at hypertensive doses (Wang et al. 2006; Hilgers et al. 2007). But in contrast, another study in rats found that contractile responsiveness to phenylephrine was significantly increased in second‐order mesenteric resistance arteries isolated from rats after 14 days of high Ang II (Giachini et al. 2011). However, second‐order rat mesenteric arteries are obviously larger than rat third‐ and fourth‐order mesenteric arteries. And it has been reported that as muscular arteries in mesenteric and other vascular beds decrease in size the density of their sympathetic innervation increases, reaching a maximum in the smallest ones (Burnstock 1975; Cowen and Burnstock 1980). Therefore, the ability of Ang II to directly increase alpha adrenergic receptor expression and contractile function of arterial smooth muscle may not be opposed as much by increased sympathetic neural traffic in rat second‐order as compared to third‐ and fourth‐order branches of the same vasculature.

Finally, to our knowledge there has only been one previous attempt to evaluate contractile responsiveness to norepinephrine in the fully intact mesenteric circulation of the rat after long‐term infusion of a hypertensive dose of Ang II (Abraham and Simon 1994). In that study, norepinephrine was injected locally into the isolated pump‐perfused mesenteric circulation and failed to exert different perfusion pressor responses in the Ang II‐treated versus control rats. As such, pressor responses were most likely dependent on norepinephrine‐induced contraction of the more highly innervated resistance vessels; this result is consistent with our findings and related hypothesis.

### Infusion of high Ang II and impaired vascular reactivity to nitric oxide

Another novel finding from the first experiment of this study was the impairment in endothelium‐independent relaxation to the NO donor, nitroprusside, specifically in our intact aortic tissue rings after the 14‐day infusion of the high Ang II (Fig. 4A) but with no corresponding decrease in endothelium‐dependent relaxation to ACh in the same rings (Fig. 3A). There are other reports of such impaired relaxation to nitroprusside and other NO donors, again specifically in aorta, after similar infusions of high Ang II to rats (Rajagopalan et al. 1996; D'Uscio et al. 1998; Mollnau et al. 2002), plus several studies of two possible mechanisms which likely explain it. High Ang II has been shown to decrease expression and related sensitivity to NO of the cGMP‐producing enzyme guanylyl cyclase and to increase expression of certain phosphodiesterases that inactivate cGMP in arterial smooth muscle, again mostly from aorta (Jacke et al. 2000; Mollnau et al. 2002; Kim et al. 2005; Giachini et al. 2011; Crassous et al. 2012). Both of these actions decrease availability of cGMP and its ability to mediate NO‐induced relaxation of such smooth muscle. But that does not explain why we did not see decreased ACh‐induced relaxation in the same aortic tissue in which our high Ang II decreased NO‐donor‐related relaxation. ACh can stimulate release of multiple relaxing factors from the arterial endothelium [e.g., NO, prostacyclin and endothelium‐derived hyperpolarizing factor (EDHF)], but in aorta and other large conduit arteries the most abundant is NO (Garland et al. 1995; Shimokawa et al. 1996; Woodman et al. 2000; Luksha et al. 2009). Thus, the ACh‐induced relaxation of our aortic ring preparations from our high Ang II‐treated rats should have at least appeared to be impaired; that is, unless ACh was somehow able to stimulate more than normal release of endothelial NO itself sufficient enough to overcome the decreased smooth muscle responsiveness to NO or was able to stimulate greater release of one or more of the other non‐NO‐relaxing factors from the endothelium of those same preparations.

It was not feasible to address these multiple possibilities in this study. We suspect the most likely is an increased release (and/or action) of EDHF for the following reason. According to one recent review (Luksha et al. 2009), when endothelial NO synthesis and/or its actions are compromised in certain forms of vascular disease (including some forms of hypertension), there may be a compensatory increase in the expression of EDHF. This form of compensation has already been seen in aorta of rats made diabetic and hypercholesterolemic over a period of 8 weeks (Malakul et al. 2008). Our results would suggest that such a mechanism was active in our aortic tissues as well over the 14‐day high dose of Ang II infusion that we employed, enhancing aortic endothelial expression of EDHF enough to completely compensate for the decreased NO function that we observed in the same tissue. Future efforts will be necessary to address this possibility.

### Infusion of low Ang II and unaltered vascular reactivity

Studies by Xue et al. (2012a), Clayton et al. (2014) and their colleagues have shown that systemic administration of a low, nonpressor dose of Ang II (10 ng/(kg·min), subcutaneously) for 7 days (followed by a 7‐day delay) markedly enhanced (i.e., sensitized) the hypertensive response elicited by subsequent treatment with either a slow‐pressor dose of Ang II or a high salt intake. Accompanying the sensitized increases in blood pressure were molecular changes in the CNS, suggesting that neuroplasticity is likely to contribute to the mediation of the enhanced hypertensive response (Johnson et al. 2015). But although sustained changes in the CNS alone may be sufficient to account for maintenance of the sensitized hypertensive response, it is possible that systemic alterations, such as increased contractile and/or impaired relaxant vascular reactivity, also contribute to the enhanced blood pressure response. The second experiment of this study investigating the effects of the 10 ng/(kg·min) Ang II treatment on vascular reactivity followed a previously established protocol (Xue et al. 2012a; Johnson et al. 2015) to test if a treatment sufficient to sensitize the hypertensive response also produced sustained changes in vascular reactivity. Our results failed to find any such changes in either thoracic aorta, mesenteric resistance vessels or the proximal portion of the caudal artery following this nonpressor sensitizing dose of Ang II. Although these findings do not prove that there are no changes in vascular function involved in mediating sensitization of the hypertensive response, the results do tend to rule out involvement of some of the most likely vascular contributors.

## Conclusions

Our results provide a new perspective on the capacity of long‐term infusions of pressor doses of Ang II to alter the expression of alpha‐adrenoceptors and thereby change their contractile function in smooth muscle of peripheral arteries. The effect of Ang II on adrenoceptors depends importantly on the amount of sympathetic innervation each vessel receives. The findings indicate that Ang II can enhance norepinephrine‐elicited vasoconstrictor properties in arteries with little or no sympathetic innervation, but not in those with dense innervation. The results also add a new perspective to the current interest in the capacity of pressor doses of Ang II to alter expression and function of NO and non‐NO relaxant substances in the arterial wall, independent of its ability to induce hypertension. Our results suggest that such Ang II may enhance endothelial release of and/or smooth muscle responsiveness to non‐NO relaxant factors in a manner that compensates for its impairment of NO‐dependent relaxation.

## Conflict of Interest

None declared.
